# A Novel Capacitive Measurement Device for Longitudinal Monitoring of Bone Fracture Healing

**DOI:** 10.3390/s21196694

**Published:** 2021-10-08

**Authors:** Angela Sorriento, Marcello Chiurazzi, Luca Fabbri, Michelangelo Scaglione, Paolo Dario, Gastone Ciuti

**Affiliations:** 1 The BioRobotics Institute, Scuola Superiore Sant’Anna, 56025 Pisa, Italy; paolo.dario@santannapisa.it (P.D.); gastone.ciuti@santannapisa.it (G.C.); 2 Department of Excellence in Robotics & AI, Scuola Superiore Sant’Anna, 56127 Pisa, Italy; 3 Orthopaedic and Traumatology Department, Pisa University Hospital, 56126 Pisa, Italy; lucafabbri8@gmail.com (L.F.); michelangelo.scaglione@gmail.com (M.S.); 4 Department of Translational Research on New Technologies in Medicine and Surgery, University of Pisa, 56126 Pisa, Italy

**Keywords:** longitudinal monitoring, biomechanics, bone fracture healing, external fixators, measurement device, capacitive-based sensor

## Abstract

The healing process of surgically-stabilised long bone fractures depends on two main factors: (a) the assessment of implant stability, and (b) the knowledge of bone callus stiffness. Currently, X-rays are the main diagnostic tool used for the assessment of bone fractures. However, they are considered unsafe, and the interpretation of the clinical results is highly subjective, depending on the clinician’s experience. Hence, there is the need for objective, non-invasive and repeatable methods to allow a longitudinal assessment of implant stability and bone callus stiffness. In this work, we propose a compact and scalable system, based on capacitive sensor technology, able to measure, quantitatively, the relative pins displacements in bone fractures treated with external fixators. The measurement device proved to be easily integrable with the external fixator pins. Smart arrangements of the sensor units were exploited to discriminate relative movements of the external pins in the 3D space with a resolution of 0.5 mm and 0.5°. The proposed capacitive technology was able to detect all of the expected movements of the external pins in the 3D space, providing information on implant stability and bone callus stiffness.

## 1. Introduction

Fracture healing is a complex and dynamic process, which involves both biological and mechanical aspects. About six million fractures occur annually in the United States, and 5 to 10% of these fractures proceed to a delayed union or a non-union [1]. Generally, a non-union fracture occurs if the reparative processes end before the bone union takes place. The biological risk of non-union depends on the fracture severity and location, i.e., highest non-union rates are associated with scaphoid (15.5%), followed by tibia and fibula (14%), and femur (13.9%) [2].

External fixators are widely used for the stabilization of long bone fractures [3] with precise indications. Usually, pins are placed on both sides of the fracture and bars are attached to the pins by using clamps. The intramedullary nail is associated with fewer complications [4], but also has many contraindications [5], and therefore the external treatment is often preferred. Indeed, the positioning of an external fixator is faster and simpler [6] and, furthermore, it also allows the dynamic adjustment of the degree of mechanical rigidity of the implant during the healing process. Moreover, external fixators have the advantage of being outside the body, allowing an objective evaluation without further surgical procedures. Successful treatment with an external fixator is strongly dependent on the identification of the correct timing for its removal. Premature removal of the fixator can lead to bone refracture, resulting in additional surgical procedures and an extended period of hospitalisation. On the other hand, delayed removal of the implant leads to an unnecessarily prolonged treatment time [7]. Hence, prevention and an adequate healing assessment are essential factors for both the patient’s well-being and to reduce the costs associated with surgical re-interventions and follow-ups.

Currently, radiographic and clinical examination are the most common tools used to monitor bone healing; however, these are subjective, operator-dependent, and non-quantitative methods. Hence, there is a need for a reliable and objective method able to quantitatively establish the state of bone health, measuring: (a) the degree of mechanical stabilization of the bone implant, and (b) an objective endpoint of healing. The monitoring of the implant’s stability may avoid further surgical interventions related to refracture and/or non-union problems. On the other hand, a precise knowledge of the stages and endpoint of healing may allow the formulation of patient-specific rehabilitation protocols.

### 1.1. State-of-the-Art of Measurement Technologies

Many researchers proposed alternative methods for monitoring the bone fracture healing, as reviewed in [1,8,9,10]. In particular, mechanical assessment tools are based on the concept that bone callus stiffness increases from the early phases of callus formation to complete union, representing a useful index of healing. Vibrational testing has been proposed to evaluate the mechanical properties of the bone–external fixator system, evaluating its resonant response to an excitation input signal [7,11,12]. However, this kind of approach suffers from metallic implant interference, and skin and soft tissue interference on the mechanical wave propagation; moreover, different studies are in disagreement on the definition of the healed fracture as its vibrational response [8]. On the other hand, direct and/or indirect biomechanical tests have been performed in research to evaluate the mechanical behaviour of the system along the healing process. As direct methods (i.e., the assessment of the fracture stiffness by measuring the deflection at the fracture site [9]) have the major disadvantage of removing the external fixator for each measurement, the indirect approach (i.e., the measurement of the strain through an external fixation [1]) has been investigated more extensively. In indirect measurement approaches, the strain of the implant is evaluated against the healing time. Since the deformation of the frame is inversely proportional to the callus stiffness, information on the state of the bone can be obtained by monitoring the level of deformation on the external fixator [13]. Only a few studies in the past have evaluated the displacement of the external fixator components as a mean for monitoring fracture healing. However, most of these studies focused only on the evaluation of the mechanical performance of the implant depending on the different frame configurations that were used [14,15,16,17], without considering the development of bone callus. Seide et al. [18] used a hexapod external fixator embedded with force sensors to measure 3D external loads. This measurement device gave results in good agreement with the healing time, even if the system appeared cumbersome and lacked portability. Grasa et al. [19] also sensorized an external fixator using a set of strain gauges to detect the bending loads on two different planes. The system was validated on animal models by recording the load transmission through the fixator during the gait cycle and over the healing time. However, as assessed by the authors, the results should be evaluated with caution, considering the high variability among the animals. Burny et al. [13] highlighted the importance of considering the presence of bone callus as it can cause variations in load transmission. Authors covered the rods of a Hoffmann^®^ external fixator (Striker Corp., Kalamazoo, MI, USA) with strain gauges in order to monitor changes in the torque applied to the rods and the moments at the articulations. The analysis was made for different configuration of the external fixator, using springs of different stiffness to simulate bone callus healing. In 1983, Beaupre et al. [20] analysed the change in pins displacements, also taking into account different degrees of bone callus stiffness using a dial gauge integrated with an external fixator. Recently, Di Puccio et al. [21] measured the load transfer process between the external fixator and the fractured bone by means of strain gauges; this study allowed us to identify the optimum position of the strain gauges on the fixator to measure the load distribution during healing.

### 1.2. Beyond the State-of-the-Art and Organization of the Paper

To summarise, all the previous studies employed, primarily, strain gauges or dial gauges in order to measure the implant deformation over the healing time. However, strain gauges can only provide a measurement on a single axis and, therefore, several sensors have to be used in order to detect the translation on each axis; this consistently increases complexity and costs of the measurement system. On the other hand, dial gauges have the advantage of being cheaper, although they can only provide linear measurements.

In this study, we propose a low-cost and customised solution composed of a 2D matrix of capacitive sensors that are able to detect both the relative translation and the rotations of the external fixator pins, as an index for determining the bone’s healing status. Indeed, capacitive sensors are cheap and easily scalable/adaptable to any non-regular surface. In particular, we decided to record the displacements of the pins, as they present the advantage of being in direct contact with the bone fragments: hence, if some movements occur at the bone level, they are directly transferred outside through the pins. The external fixator deforms when the bone is subjected to an external load (as during the weight bearing), and such deformation decreases when the bone callus stiffness increases. Usually, patients are encouraged to bear weight on the injured limb, as much as the pain allows it, from day one, in order to stimulate bone regeneration, while leading to a deformation of the external frame. Indeed, it has been shown that interfragmentary movements can significantly enhance callus formation [22]. On the other hand, if the callus is not stiff enough, an excessive external load can lead to plastic deformation of the fixator, with the consequent need of surgical re-intervention [9,14]. Hence, after each weight bearing exercise, a measurement of the degree of deformation on the external frame is always needed to assess the stability of the external implant.

The authors decided to focus such study on tibia fractures since, as mentioned above, they are associated with the highest non-union rates. Moreover, a fracture which involves the lower limbs forces the patient not to move for a long period of time, causing discomfort that lasts for a long period of time in the case of non-union.

In this article, we first implemented and tested a finite element model (FEM) of a bone fracture model, stabilised with a Hoffman II^®^ external fixator (Striker Corp., Kalamazoo, MI, USA) in the standard configuration used for tibia fractures (see Section 2.1). In Section 2.2, this model was used to systematically analyse the pins displacements due to external loads for each condition of callus stiffness. Finally, in Section 2.3, a novel capacitive technology was developed and integrated, as part of a novel measurement device, to discriminate the expected displacements of the external fixator pins during the healing time (Section 2.3.1 and Section 2.3.2). The results support the use of such capacitive sensor technology for quantitative and even remote assessment of external fixator stability and bone fracture healing. This study represents a first preliminary step toward a future in-vivo application of such a sensorized device.

## 2. Materials and Methods

**Figure 1:**
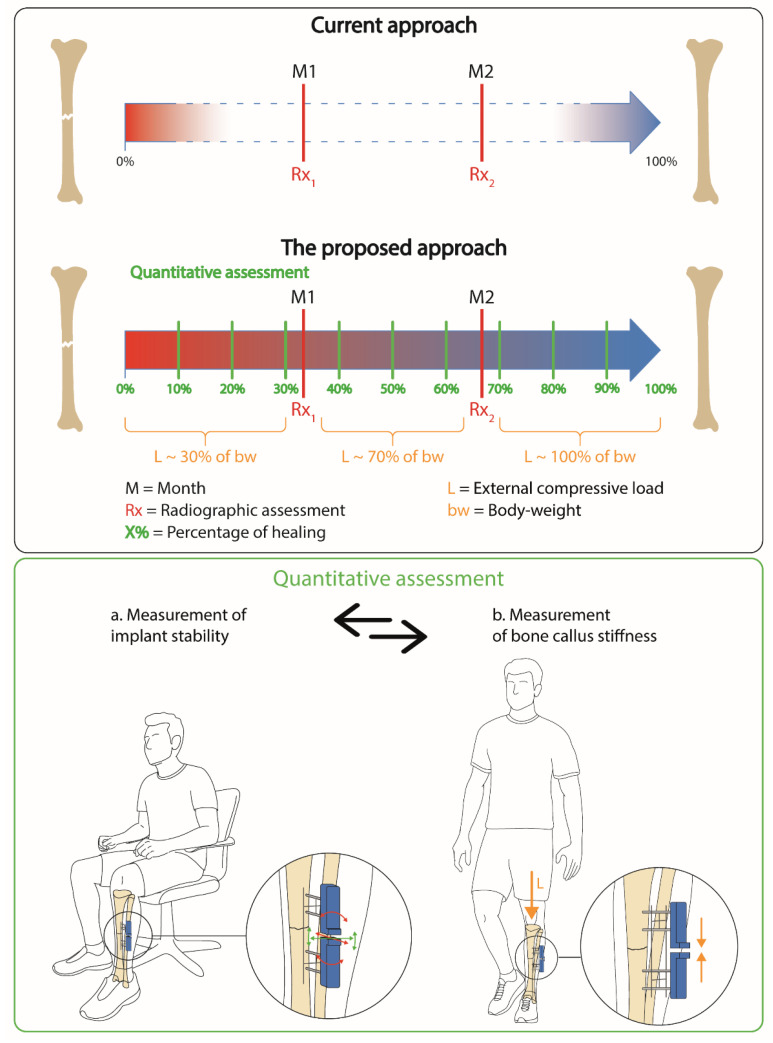
Management of bone fracture assessment: current approach versus our proposal.

The aim of this study is to implement a novel measurement methodology and technology (see Figure 1, top panel) allowing patient-specific and quantitative assessment of bone healing, based on the development of a compact sensorized instrument integrated into the external fixators (see Figure 1, bottom panel). The capacitive technology was developed to provide a twofold monitoring, i.e., (a) the measurement of implant stability, and (b) the measurement of bone callus stiffness. The measurement of implant stability (see Figure 1, bottom panel a) is necessary to verify that the implant is always in the original position and that no plastic deformations occur due to previous weight bearing exercises. After checking the stability of the whole system, a measurement of the bone callus stiffness (see Figure 1, bottom panel b), at increasing external compressive loads (namely L in Figure 1, top panel), can be performed to derive an objective metric of healing. From a future clinical perspective, the measurements (a) and (b) (Figure 1, bottom panel) can be used as part of a cyclic and repeatable assessment over the healing period, with the aim of supporting the current diagnosis approach, providing longitudinal and quantitative information on both implant stability and the healing percentage of the bone, which is useful in developing patient-specific rehabilitation protocols. It is worth highlighting that we here provide an example of bone fracture management (according to the clinical indication provided by co-authors), but the procedure could be slightly different depending on the type of fracture and on the clinician’s preferences.

### 2.1. Design and Validation of a Bone–External Fixator Model

A system including the bone and the external fixator was modelled to investigate its mechanical behaviour. We decided to analyse the deformation of the Hoffmann II^®^ external fixator, as it is a widely used orthopaedic implant in surgery, in the standard and clinically relevant bi-lateral configuration used in the case of a tibia fracture [23]. The bi-lateral frame was connected to the bone by means of four stainless steel self-drilling pins (5 mm in diameter and 150 mm in length). The aluminium alloy clamps, with a maximum of 5 pin holes, were connected to the pins at a distance of 45 mm (indicated by the clinical co-authors as a typical distance in the clinical practice). Two rods (8 mm in diameter and 250 mm in length) made from Vectran^®^ (Kuraray Co., Ltd., Tokyo, Japan), coated with carbon fibre, were applied laterally. An additional carbon fibre bar (8 mm in diameter and 200 mm in length) was placed transversally to further increase the rigidity of the frame in case of an absent callus.

**Figure 2:**
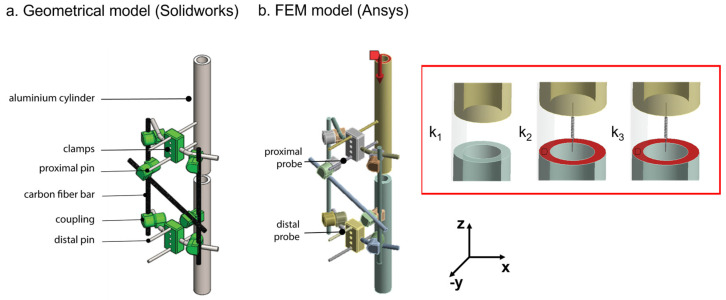
Model of the bone-external fixator system: (**a**) geometrical representation; (**b**) FEM model.

First, geometrical modelling of the analysed Hoffmann II^®^ external fixatorconfiguration was implemented in mechanical computer-aided design (CAD) software, Solidworks^®^ (Waltham, MA, USA), as reported in Figure 2a. Based on such geometry, a FEM model was performed using a simulation software Ansys^®^ (Canonsburg, PA, USA), as shown in Figure 2b.

We performed a FEM model in Ansys^®^ using the structural mechanics module to qualitatively evaluate the behaviour of the bone–external fixator system under compressive loads. The mathematical equation on which this mechanism is based is the stress–strain relationship:

*σ* = *E* × *ε* (1)

where *σ* is the stress vector, *E* is the stress–strain matrix, and *ε* is the strain vector. In our specific case, the materials were modelled as isotropic and linear and, hence, information on the Young’s modulus and Poisson’s ratio was given for each material. Further information on the theoretical bases for the mechanical module of Ansys^®^ can be found in the relevant tutorial [24].

The fractured bone was modelled as an aluminium cylinder, as suggested in a previous work to reduce the variability related to geometry and mechanical properties [14]. The aluminium tube had a 30 mm outer diameter, a 20 mm inner diameter, and a total length of 500 mm. A 20 mm gap was left at the fracture site and the bone callus was represented by springs of different rigidity: k_1_ = 0 N/mm, k_2_ = 0.153 N/mm and k_3_ = 0.460 N/mm. We used springs with increasing rigidity to simulate increasing bone callus stiffness, while the values of springs were chosen to be feasible with benchtop tests and in accordance with previous works [13]. The behaviour of each component of the external fixator was assumed to be isotropic, linear, and homogenous, based on data provided by the datasheet [23], and according to similar studies observed in the literature [21,24]. The experimental validation supported the validity of these assumptions for the purposes of this study. Bonded-type contacts were used to simulate the joints between the elements of the external fixator. At the end of the proximal bone segment, an axial compressive force was applied in the longitudinal direction (*z*-axis), as reported in Figure 2b, red arrow). A displacement constraint, allowing rotations only in the transversal plane (xy-plane in Figure 2), was applied at the end of the distal bone to simulate the real movement of the system subjected to a compressive load.

When the patient is standing up, the frame must support part of the patient’s weight and, therefore, it is subjected to axial compression. Unfortunately, there are no available data regarding the precise level of loads applied to the frame in clinical applications. However, the frame is not expected to sustain the full load before partial bone healing occurs. For this reason, we chose to apply increasing loads from 0 to 300 N with a constant step size of 50 N, according to data found in the scientific literature [13,14,20], and in accordance with the medical co-authors’ evidence and considerations. The deformation was recorded in the FEM simulation placing two probes: (1) one on the proximal pin, and (2) the other on the distal pin (as shown in Figure 2b). The pins closest to the fracture site were chosen as target pins due to their more consistent displacement.

**Figure 3:**
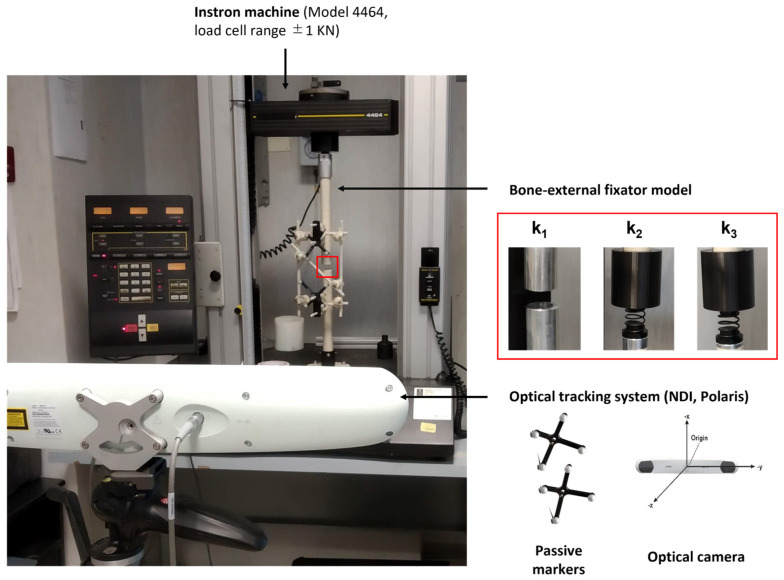
Experimental setup for FEM model validation.

Experimental tests were performed to validate the model described above, as shown in Figure 3.

The elements of the external fixator were assembled, tightening the joints of the frame using a controlled torque wrench with 11 Nm, in order to guarantee an elastic behaviour of the external frame, as recommended in [14]. The axial loads were applied to the analysed configuration of the fixator using a universal material testing machine (Instron, model 4464, Norwood, MA, USA), with a load cell in the range of ±1 kN. Both sides of the system were fixed to the Instron machine, avoiding undesired oscillations. An optical tracking system (Polaris Spectra, Northern Digital Inc. (NDI), Waterloo, ON, Canada) with an accuracy of 0.25 mm [25] was used to record the displacement of each target pin on either side of the gap fracture. In particular, a tool including more than three markers (i.e., three markers needed to extrapolate the pose plus one marker for redundancy [26]) was fixed on the target pin and tracked by the optical camera through a dedicated software interface, properly developed by the company. The aluminium cylinder was covered using paper tape to avoid undesired reflections of the optical light and thus possible measurement errors. The bone callus was reproduced, as in the simulation, using springs with different stiffnesses, which were placed in the fracture gap using an ad hoc 3D printed frame. Three stiffness conditions were tested, as mentioned, i.e., k_1_ = 0 N/mm, k_2_ = 0.153 N/mm and k_3_ = 0.460 N/mm, and three compressive tests for each stiffness were performed. During each test, the intensity of the load was varied from 0 to 300 N using a velocity of 5 mm/min and a step size of 50 N. At each step, the Instron machine was stopped to record the coordinates of the pins in static conditions by using the optical tracking system. For each load, a total of 400 frames were recorded to average possible localization errors. Data acquired with the optical tracker were processed off-line with Matlab^®^ R2018b (MathWorks Inc., Natick, MA, USA). At each loading step, the Cartesian coordinates (x, y, z) of the proximal and distal pins were handled to obtain the Euclidean distance. Then, the mean of the three acquisitions was calculated to extract one single value for each load. The same procedure was applied for all the stiffness conditions. Similarly, data from the simulation were processed, obtaining the Euclidean distance between the proximal and distal pins for each load and for each stiffness of the springs. Finally, the target measures from the simulation and from the experiment were compared, calculating the relative percentage error.

### 2.2. Pins Displacements during the Simulated Bone Healing

After validation and confirmation of the appropriateness of the simulated environment (results are presented in Section 3.1), the FEM model was used to calculate the pins displacements for different healing phases and for different external loads. According to the literature, the complex phenomena involved in bone healing can be commonly described as a 4-phase model [27]: (1) an inflammation phase, (2) soft callus formation (cartilage form), (3) hard callus formation (woven bone), and (4) bone remodelling. The inflammatory phase dominates the very early stage of healing (about 2 days after fracture). The soft and hard callus stages represent the reparative phase, which occurs from 3 to 6 weeks after the fracture occurred. Finally, the remodelling phase can take from a few months to years [28,29]. However, to avoid complications in the bone growth, it is important to intervene in the early stages of healing (up to the reparative phase), as in the remodelling phase, the bone is already formed, and adjustments cannot be assured.

It was demonstrated that the ability of a patient to bear weight on the fractured limb increases with the time post-fracture and, hence, with bone callus stiffness [30]. Since the capacity to sustain external loads is completely subjective, there are not clear and standard guidelines in the literature about the load levels to apply during the healing time. Hence, clinical specifications were provided by our co-authors, who are surgeons experienced in the orthopaedic field. Usually, the patient is able to sustain about the 30% of his/her body weight until the third week after fracture and, during the reparative phase (3 to 6 weeks), the patient may support up to 70% of his/her body weight, succeeding in supporting his/her body weight entirely in the remodelling phase.

Considering the above-mentioned considerations of the clinicians, we calculated in the simulation the pins displacements for three ranges of callus stiffness, which reproduced three phases of healing: (1) 0–10 MPa, (2) 10–1000 MPa, and (3) 1000–6000 MPa [31]. The first range of stiffness simulated the inflammation/soft-callus phase, in which the patient is able to support around 30% of their body weight. The second range of stiffness reproduced the soft/hard-callus phase, in which the patient may sustain around 70% of his body weight. Finally, the third range of stiffness replicated the remodelling phase, in which the patient reaches full weight bearing capacity. A body weight of 70 kg was considered for this analysis, as it represents the average weight of the European population [32]. All the clinical indications are reported in Table 1. For each callus stiffness, we applied as the external load an increasing percentage of the body weight (from 0% to 100%), and we derived the Euclidean distance between the proximal and distal pins of the external frame.

### 2.3. Design of a Capacitive Sensor Technology to Discriminate Pins Movements

In order to monitor the pins displacements, the bone–external fixator system was endowed with a sensorization module capable of detecting such displacements. In this regard, capacitive sensors were employed in a parallel plate configuration, to provide pre-contact information (0–20 mm distance), as already described in [33].

Such configuration exploits a parallel plate capacitor, where the sensors are made up of two plates placed at a specific distance. When, due to mechanical stresses, the relative distance between the two plates changes, the variation in capacitance is measured as:

(2) $$C=\varepsilon_{0}\cdot \;\frac{A}{d}\;\;$$

where *ε_0_* is the dielectric of the propagation medium (i.e., air in our prototype), whereas *A* is the area of the plates and *d*, being the relative distance among them, is the variable we indirectly compute throughout the capacitance variation.

Although this configuration cannot provide a highly sensitive range, such an approach offers a good resolution. Parallel plate capacitors are mainly employed to provide pressure and pre-contact information. Electrostatics is the subfield of electromagnetics describing an electric field caused by static charges. In this case, the relationship with the electric field *E* is:

(3) $$\nabla E=\frac{\rho }{\varepsilon_{0}}$$

where *E* is the electric field, *ρ* is the space charge density and *ε*_0_ is the permittivity of the free space. The charge–field relationship requires that the electric field is irrotational:

∇ × *E* = 0 (4)

For an irrotational field, the definition of the electric potential is:

−∇*V* = *E* (5)

For more details, please refer to [34]. Based on these equations, we implemented in Comsol Multiphysics^®^ (version 5.3a; Comsol Inc., Stockholm, Sweden) a parallel plate configuration, in which one plate was grounded (*V* = 0), while the deposition of charge was read on the other plate; each simulation applies a pre-compiled constant mesh and time step.

**Figure 4:**
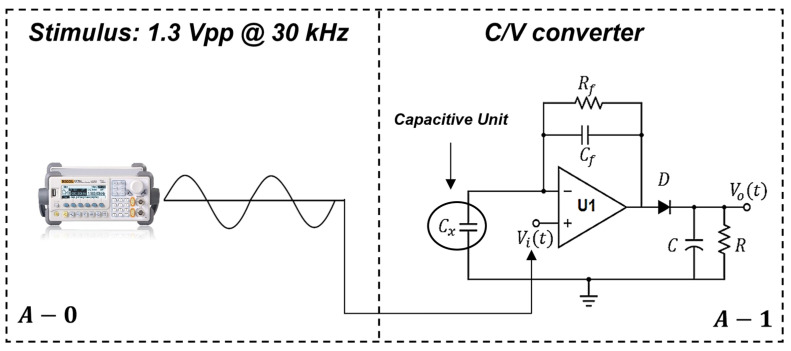
Electronic characterization circuit for the single capacitive sensor unit (*C_x_*).

Physical parameters of the sensitive unit and its optimal positioning in the external fixator were chosen computing a Nelder-Mead optimization model [31] implemented in Comsol Multiphysics^®^. Considering key control variables, i.e., (1) the sensor area and (2) the ratio between electrode dimensions, the model was used for optimizing a specific cost function represented by the electrical field norm. Different smart arrangements of this sensor unit were implemented to provide both (a) the measurement of implant stability, and (b) the measurement of bone callus stiffness. The electronic characterization circuit for the single capacitive sensor unit was developed and depicted in Figure 4. A signal generator is used to provide a 1.3 *Vpp* sinusoidal signal at 30 kHz–0° (see Figure 4, panel A-0). Through the virtual short-circuit of U1 (see Figure 4, panel A-1), one electrode of the reading sensor (Figure 4 panel A-1) is stimulated, whereas the second electrode is grounded. The design of the electronic characterization circuit is conceived in order to be compact in size and limited in terms of power consumption. In Figure 4 (panel A-1), a C/V converter (ADA4610-1ARZ, Analog Devices Inc., Norwood, MA, USA) is used to amplify and convert the variable capacitance into a voltage signal. The stimulus frequency is limited by both the maximum analogue input voltage of the ADC and the power supply of the ADA4610 C/V converter. Moreover, according to ICNIRP guidelines [30], the chosen frequency (30 kHz) is not dangerous for the patient, even in cases of long-term exposure. The signal is half-rectified by using a diode (D) and then filtered using a band-pass filter. This last step was implemented, via software, in LabVIEW^®^ programming language (National Instrument Corp., Austin, TX, USA), where the signals were acquired using a NI DAQmx 6363 board with a sampling rate of 150 kHz (in order to respect the Nyquist criterion).

#### 2.3.1. Measurement of Implant Stability

**Figure 5:**
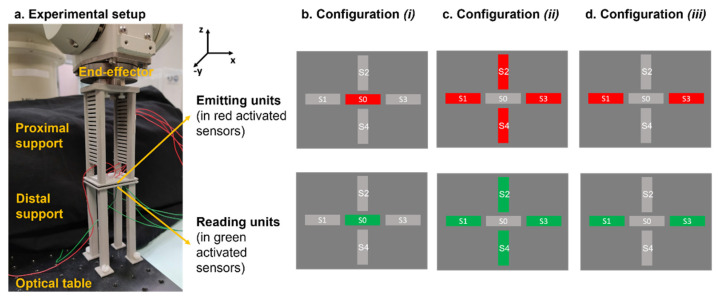
Experimental setup for characterizing the capacitive sensor technology.

As already assessed, it is essential that the whole system, consisting of the bone and the sensorized external fixator, does not undergo undesired movements, which are not correlated with the bone healing process. The external fixator does not guarantee a completely rigid stabilisation and thus small linear and angular movements can occur in all the directions of the 3D space. Thanks to the clinical specifications, provided by the medical co-authors, we learnt that a linear movement in the range of ±1 mm and an angular displacement in the range of ±1° can occur in the clinical practice. Since we want to derive an objective percentage of healing with respect to the specific patient and implant, the results on a certain day have to be related to those of the previous days, and hence unwanted movements of the external fixator should be detected. One single capacitive unit is not sufficient to discriminate any linear and angular movements in the transversal plane (see results in Figure S2 of the Supplementary Materials). Hence, to detect all six degrees of freedom (i.e., displacement along the three axes: T_x_, T_y_, and T_z_, and rotations around the three axes: R_x_, R_y_, and R_z_), a total of 5 sensor units were embedded in the measurement device: 4 units arranged laterally with respect to the central unit (as depicted in Figure 5b, c and d). Five conductive tapes (3M Company, Saint Paul, MN, USA) with 2D dimensions of 2.9 mm (L) and 13.4 mm (W) were attached to the distal and proximal support (see Figure 5a) with a relative distance avoiding any interference during the recording.

Experimental tests were performed in order to describe the main features (i.e., sensitivity, accuracy, precision, time of response, and resolution) of the measurement device. In particular, three different configurations were implemented and tested: (1) only S0 was activated (see Figure 5b) in order to record the longitudinal linear displacements (T_z_), (2) S1, S2, S3, S4 were simultaneously activated (see Figure 5c) to monitor linear and angular movements in the transversal plane (T_x_, T_y_, R_x_ and R_y_), and (3) a combination of sensors S1 and S3 was used (see Figure 5d) to detect angular movements around the *z*-axis (R_z_). Starting from the sensors’ configuration (position and dimension), which was optimized throughout the Nelder–Mead optimization module, the three configurations arose from several simulation tests performed in Comsol Multiphysics^®^ (Comsol Inc., Stockholm, Sweden), where the curve of response of each sensor was computed and compared in order to understand the most significative subgroups to probe according to the movements to monitor (x–y–z translation/rotation). The sensor unit employed in the experimental tests has 2D dimensions of 2.9 mm (L) and 13.4 mm (W), with an overall thickness of 0.5 mm. The proximal support of the sensors was attached at the end-effector of an anthropomorphs industrial robot (RV-3SB series, Mitsubishi Corp., Marunouchi Chiyoda-ku, Tokyo, Japan), whereas the distal support for the sensors was fixed to an optical table (see Figure 5a) to guarantee high stability.

For each repetition (i.e., 3 in total to evaluate repeatability), the end-effector of the robot performed a set of linear displacements (from −2 to 2 mm) with a step size of 0.5 mm (lineal spatial resolution) and a set of angular displacements (from −2° to 2°) with a step size of 0.5° (angular spatial resolution) in each direction in the 3D space. At each step, data from the capacitive sensors were recorded in static conditions for all three configurations using a LabVIEW^®^ programming interface (National Instrument Corp., Austin, TX, USA). Then, data were processed offline in Matlab^®^, displaying the movements to be detected by each configuration. When the recording was performed by more than one sensor simultaneously, the data from all the activated sensors were normalized for each displacement independently, creating a colour map (between 0 and 1) to better visualize and represent the response of the sensors for a given movement.

#### 2.3.2. Measurement of Bone Callus Stiffness

**Figure 6:**
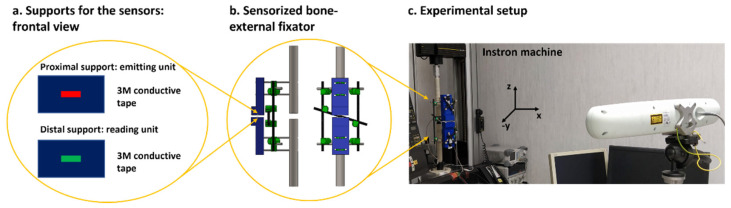
Experimental setup for testing the capacitive sensor technology.

Figure S1 of the Supplementary Materials demonstrates that the deformation of the entire frame under axial compression resulted to be mainly longitudinal. Hence, for detecting linear displacements in the longitudinal direction (*z*-axis), a single sensor unit with a rectangular geometry (L = 2.9 mm and W = 13.4 mm) was designed, implemented, and integrated onto the frontal surfaces of the two pins using two ad hoc 3D printed supports (see Figure 6a,b), which were positioned at an initial relative distance of 2 mm.

Experimental tests were performed to prove the ability of the capacitive sensor module to monitor displacements during axial compression (*z*-direction). For this purpose, the sensor technology was integrated into the experimental setup described in Section 2.1 (see Figure 6c). The stiffness condition k_1_ = 0 N/mm was chosen for this analysis, and five compressive tests were then performed. During each test, the intensity of the load was varied from 0 to 500 N using a velocity of 5 mm/min and a step size of 50 N. At each step, the Instron machine was stopped to record the movements of the pins in static conditions by using both the optical tracking system and capacitive sensor technology. Data were collected and processed in Matlab^®^, deriving the sensor curve with respect to the applied loads. Finally, the real data from the five tests were normalized and matched with the normalized data derived from the simulation, in order to validate the Comsol Multiphysics^®^ FEM model.

## 3. Results

### 3.1. Validation of the Bone-External Fixator Model

**Figure 7:**
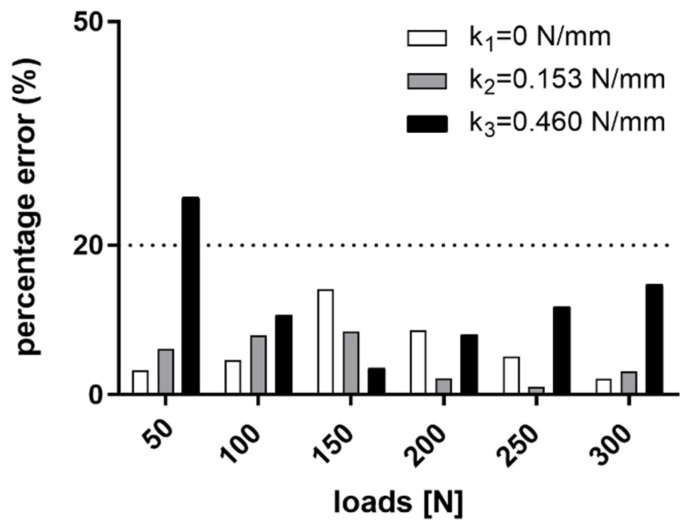
Percentage error between simulation and experimental results.

In Figure 7, the error bars between experimental data and FEM simulations are presented in terms of percentage error. A threshold of 20% is usually considered acceptable by the scientific community [35]. In our tests, the percentage error was higher than 20% (i.e., 26%) only for the very low load (i.e., 50 N) at the higher stiffness condition (i.e., k_3_ = 0.460 N/mm), where the displacements of the pins were in the order of tens of micrometres (i.e., 0.15 mm) and thus under the optical tracking system accuracy (i.e., <0.25 mm). The average percentage error is 6%, 5% and 12% for k_1_, k_2_ and k_3_, respectively, and overall is equal to 8%.

### 3.2. Typical Pins Displacements during the Simulated Bone Healing

**Figure 8:**
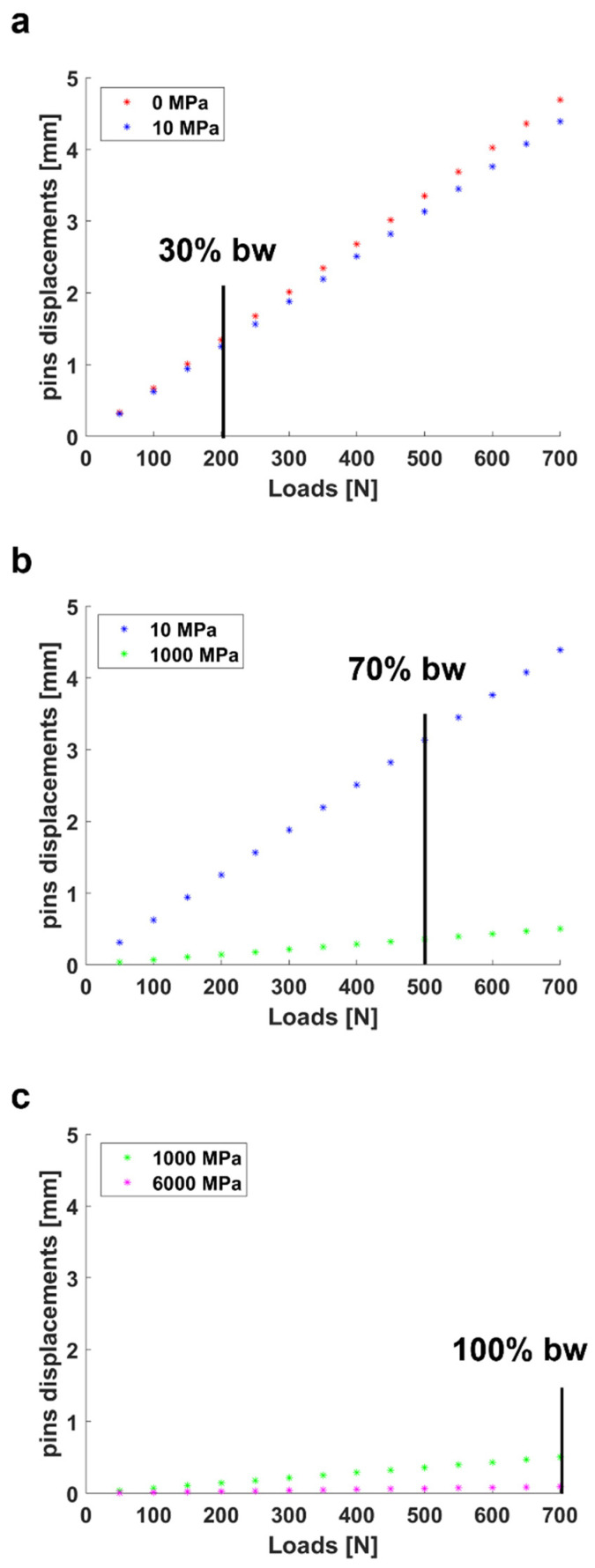
Pins displacements calculated in the simulation for: (**a**) inflammation/soft callus phase, (**b**) soft callus/hard callus phase and (**c**) remodelling phase.

Figure 8 shows the pins displacements, derived from the FEM model, due to compressive external loads and for increasing bone callus stiffness. The three main phases of healing, described in Table 1, were considered: (1) the inflammation/soft callus phase (Figure 8a), (2) the soft callus/hard callus phase (Figure 8b), and (3) the remodelling phase (Figure 8c). For each healing phase, the maximum percentage of body weight that the patient is supposed to be able to support was also indicated: (1) 30% for the first phase, (2) 70% for the second phase, and (3) 100% for the last phase.

In the third phase, the patient is supposed to be able to stand on his full weight without any problem. Indeed, the estimated movement of the pins at this stage is very limited (Figure 8c). As expected, and represented in the graphs of Figure 8, the pins displacements increased with an increase in the applied load, while it decreased with increasing bone callus stiffness. The range of pins displacements was found to be 1.25–1.34 mm for the inflammatory/soft callus phase at a load equal to 30% of the body weight, and 0.36–3.13 mm for the soft callus/hard callus phase at a load equal to 70% of the body weight.

### 3.3. Design of a Capacitive Sensor Technology to Discriminate Pins Displacements

#### 3.3.1. Measurement of the Implant Stability

**Figure 9:**
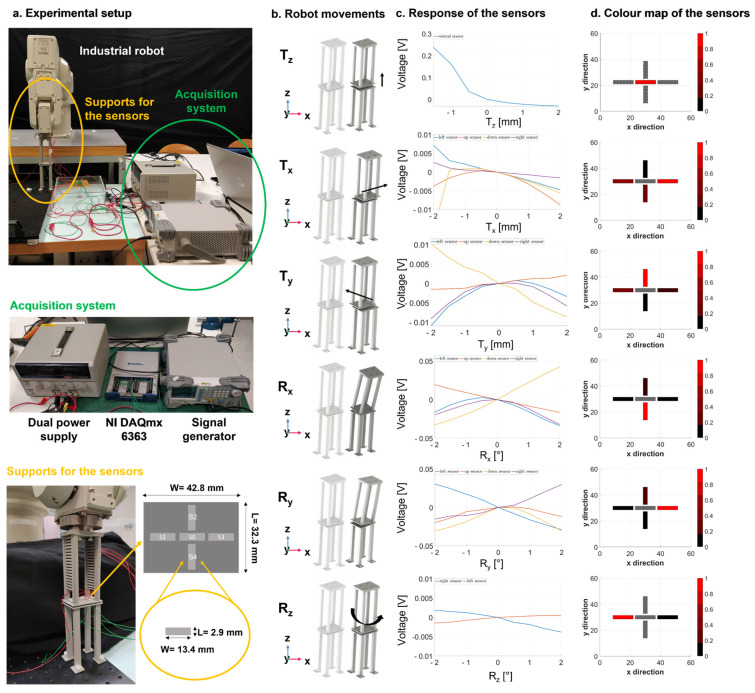
Characterization of the five-sensor architecture capacitive technology.

In Figure 9, the ability of the five-sensor architecture to detect movements in the 3D space was evaluated in three different configurations: (1) only S0 was activated to detect T_z_ (see first row of Figure 9), (2) S1, S2, S3 and S4 were activated at the same time to detect T_x_, T_y_, R_x_ and R_y_ (from second to fifth row of Figure 9), and (3) S1 and S3 were simultaneously activated to record R_z_ (last row of Figure 9). The movements performed by the robot are represented in Figure 9b), whereas the responses of each activated sensors for each performed displacement are reported in Figure 9c for the range from −2 to 2 mm and −2° to 2°, with a constant step size of 0.5 mm and 0.5°, respectively. Finally, a representative colour map for each type of displacement is shown in Figure 9d, in which the normalized responses of all the activated sensors are reported in the range [0, 1], in order to better visualize the response of the sensors. The colour maps graphically describe the sensors more involved in each type of movement. The extent of the represented movements is 2 mm for all the linear displacements and 2° for all the angular displacements. The disabled sensors are indicated in grey with a discontinuous outline. These results confirm the effectiveness of the central sensor unit in detecting linear displacement along the *z*-axis. The right and left sensors had the main role in the detection of T_x_, R_y_ and R_z_, while the top and bottom sensors were those more involved in the detection of T_y_ and R_x_.

#### 3.3.2. Measurement of the Bone stiffness

Experimental tests were performed to prove the capability of a single capacitive sensor unit to discriminate pins displacements under external compressive loads (from 0 to 500 N) in case of a callus stiffness equal to 0 (i.e., first healing phase). At the same time, the pins displacements were recorded by an optical tracking system (i.e., Polaris Spectra) as a gold-standard method.

**Figure 10:**
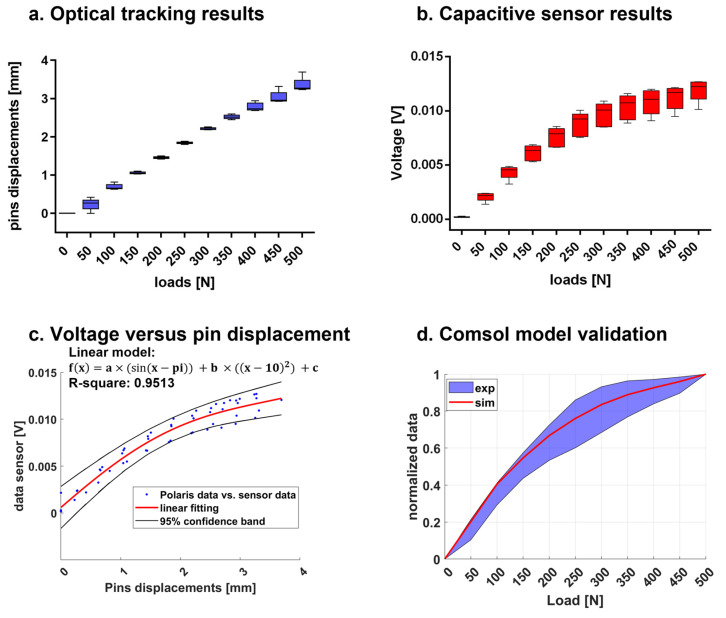
Results of the capacitive sensor technology tests.

The pins displacements recorded by the optical tracking systems are reported in Figure 10a, confirming again a linear behaviour of the pins displacements with respect to the applied loads. In Figure 10b, the capacitive sensor’s response is presented for each loading step: the exponential trend of the voltage demonstrated the ability of the capacitive module to discriminate even displacements caused by close external loads. The sensor responses were also displayed against the recorded pins displacements and fitted using a linear model with R-square equal to 0.95 (see Figure 10c). Finally, the normalized results of the capacitive sensor were compared with the normalized Comsol Multiphysics^®^ FEM results at the same loading conditions, demonstrating good matching (as reported in Figure 10d). In greater detail, the lower extremity of the violet band includes the mean data minus two times the standard deviation, while the upper extremity of the band includes the mean data plus two times the standard deviation, thus representing the real data of the three compressive tests with a 95% confidence interval. The results from the FEM simulations, shown with a red line, are entirely inside the confidence interval of the real data.

**Figure 11:**
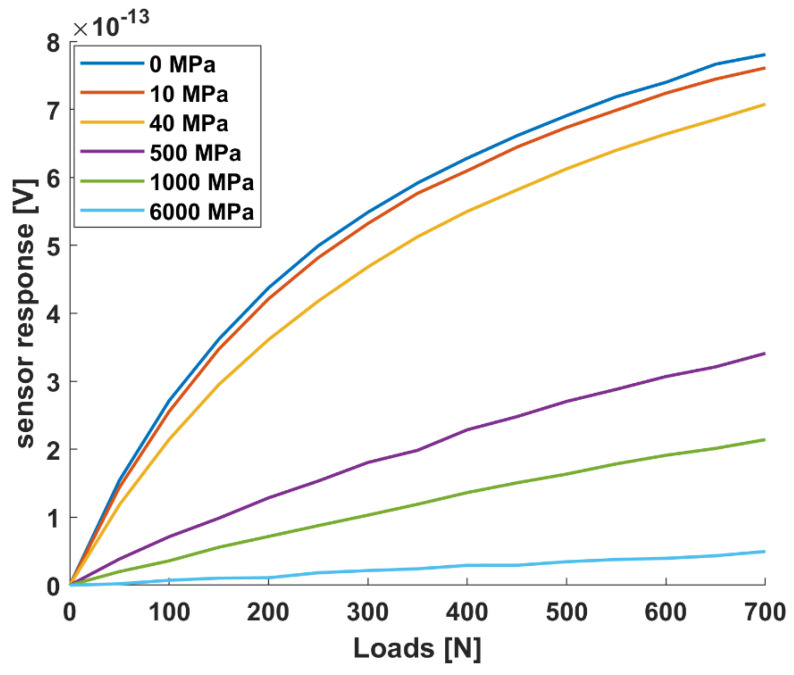
Simulated response of the single sensor unit varying the bone callus stiffness.

The validation of the Comsol Multiphysics^®^ model allowed us to calculate the sensor response for different bone callus stiffnesses, representative of each healing phase. The sensor results, in response to pins displacements derived from Ansys^®^ FEM model, are reported in Figure 11 for six representative stiffness conditions (0, 10, 40, 500, 1000 and 6000 MPa). The stiffness conditions 0, 10 and 1000 MPa correspond to the beginning of each healing phases identified in Table 1: (1) inflammatory/soft callus, (2) soft callus/hard callus, and (3) remodelling.

The results in Figure 11 indicate a well-separated sensor response for each stiffness, suggesting the possibility to discriminate pins movements associated with different healing phases. The sensor response appears slightly flattened for the highest stiffness (greater than 500 MPa), which is associated with the healing stage in which the bone is already formed.

## 4. Discussion

In this study, we developed and tested a novel low-cost and effective sensing methodology for monitoring the healing process in cases of long bone fractures treated with an external fixator. Different stabilization methods (i.e., intramedullary nailing, screwed plates, and external fixation) can be indicated for bone reduction, according to skin status, fracture type, location, and bone quality. External fixators are widely used, since they have the advantage of being flexible and have an adjustable mechanical rigidity in accordance with the natural bone growth [36]. However, external fixators have to be removed, and the identification of correct indications for their removal is fundamental. In fact, to date, there are no standard methods to objectively recognize the stages and the endpoint of healing for removing the bone implant correctly. Moreover, a precise knowledge of the endpoint of healing could enable the definition of patient-specific rehabilitation protocols.

Since the deformation of the external fixator has been demonstrated to be in inverse proportion with the bone callus stiffness [8], our scientific hypothesis seeks to obtain information on the state of the bone growth by measuring the displacement of the external frame over the healing time. The pins of the external fixator are self-drilled in the bone and are mostly external to the body, proving to be the ideal candidates for the sensorization. As the performance of the external fixation is strictly related to the arrangement and material properties of the main components, we systematically analysed, referencing the clinical standard but with a versatile approach, the mechanical behaviour of a Hoffman^®^ II external frame in the standard configuration used for tibia fractures. Indeed, our approach may also be translated to other types of external fixators, which allows access to external pins, such as uniplanar external fixators [37]. We developed and tested in Ansys^®^ a FEM model of the bone–external fixator system to investigate the relative pins displacements due to the applied compressive loads. In Figure 7, the percentage errors between the simulation values and the real values are calculated for three stiffness conditions: k_1_ = 0 N/mm, k_2_ = 0.153 N/mm and k_3_ = 0.460 N/mm and for increasing compressive loads (from 0 to 300 N with steps of 50 N). The model was verified with a percentage error lower than 20% in most of the cases, and an overall error of 8%. The error bars rose with the increasing callus stiffness, reaching a percentage error greater than 20% for the lowest loads (i.e., 50 N) at the higher stiffness condition (i.e., k_3_ = 0.460 N/mm). Indeed, as stiffness increases, the recorded pins displacements reduced, and in particular, they were under the accuracy of the optical tracking system used (i.e., <0.25 mm [26]). After the validation step, the FEM model was used to simulate the relative pins displacements for different spring stiffnesses, thus covering all the phases of healing, as shown in Figure 8. Unfortunately, a standard rehabilitation protocol is not available so far for bone fracture treatment: in most cases, the patients are encouraged to bear as much weight on the leg as pain allows. For this reason, clinical indications were provided by expert orthopaedic surgeons in the field, co-authors of this study, and an indication of the maximum supported load for each healing phase was given as a percentage of the body weight of the patient, as usually performed in orthopaedic procedures. As expected, the simulated results (Figure 8) indicate an increase in the pins displacements with an increasing external load and a decrease in the pins movements with increasing bone callus stiffness. A similar approach may have great potential in clinical practice: indeed, a FEM model may be built specifically for each patient and an estimation of the expected pins movements for each healing phase and for each external load can be obtained from such a model. If the pins displacements recorded by the sensorized external fixator are very different from those estimated by the model, it means that something wrong happened in the healing process, and thus the intervention of the physician is needed. Behind the cited advantages, it is worth mentioning that we have to consider the simulated results. They are strictly dependent on the position of the reading probe on the single pins: indeed, the movements were amplified as we moved the probe away from the fixator body. In our case, to bring the simulated results in line with our application with the embedded sensors, we placed the reading probes on the pins at the same coordinates at which the supports for the sensors were positioned in the experimental tests (see Figure 6).

Considering the described results and requirements of the study, we developed a novel sensing measurement device based on a capacitive technology which is able to provide potentially longitudinal and non-invasive/portable monitoring. The proposed sensor technology has to satisfy a double objective: (a) the monitoring of implant stability by detecting unwanted movement of the pins in the 3D space, and (b) the measurement of bone callus stiffness, which depends on the relative pins displacements under external compressive loads. The primary aim of the sensorized device is to guarantee a longitudinal and objective monitoring during the healing time. In particular, we are not interested in calculating an absolute measure of healing (i.e., the callus stiffness); instead, we want to determine a patient-specific percentage of healing over time. For this reason, the pins displacements measurement made on each patient must be related to those of the previous days, ensuring that the bone–external fixator system has not undergone any displacements not related to the healing process. Since sensors are rigidly attached to the pins, any movement of the pins can be directly transferred to the sensor supports. We found that one single capacitive module was not enough to monitor the position of the external pins in the 3D space (see Figure S2 of the Supplementary Materials). In this regard, a smart configuration of five sensors was implemented and integrated in the system, obtaining a total surface of 32.3 mm × 42.8 mm. Instead of activating all five electrodes at the same time, we found it more convenient to test three different configurations of activation, which are able to detect up to six degrees of freedom. When only the central module (S0) was activated, the displacement *T_z_* was accurately determined, with a resolution of 0.5 mm, as described in Figure 9 (first row). Similarly, the four sensors (S1, S2, S3, and S4) worked simultaneously to detect the movements *T_x_, T_y_, R_x_* and *R_y_* successfully (see Figure 9, second row–fifth row). Finally, the combination of the two lateral sensors (S1 and S3) proved to be the most suitable units to identify the rotations around the longitudinal axis (*R_z_*), as reported in Figure 9 (last row). Usually, movements of the pins not related to the healing process are in the range ±1 mm and ±1°. The proposed technology was able to discriminate movements with a resolution of 0.5 mm and 0.5°, satisfying the clinical requirements.

As the deformation of the whole frame under compressive loads was demonstrated to be axial with good approximation (see Figure S1 of the Supplementary Materials), a single sensing module centred on the sensor support proved to be enough to monitor the relative pins displacements. Experimental tests were performed to evaluate the pins displacements of the sensorized external fixator with a callus stiffness equal to k_1_ = 0 N/mm under increasing compressive loads (from 0 to 500 N) with intervals of 50 N. Figure 10b presents the sensor responses in terms of voltage at each loading step, showing an exponential trend. Such behaviour, consistent with the scientific literature of parallel plate capacitive sensors [33], can be considered advantageous, since pins displacements due to very close loads (step of 50 N) can be easily detected and discriminated. A linear regression, validated with R2 statistic equal to 0.9513, was found when we correlated the voltage and pins displacements recorded by the optical tracking system, as described in Figure 10c. Indeed, a reliable model correlating voltage and pins displacements allows us to determine the movements of the pins by reading the voltage response. All the considerations on the geometry, dimensions and positions of the sensor module were made by computing an optimization model implemented in Comsol Multiphysics^®^. In our experiments, we recreated the same conditions of the simulation, and we compared the normalized results obtained with the Comsol Multiphysics^®^ model with respect to the normalized results from the experiments, achieving a good matching (as depicted in Figure 10d). The maximum percentage error between the real and the simulated sensor curve of response was 27% (at 50 N), proving the goodness of the implemented model, which was thus used as a predictive model for simulating other conditions (i.e., different stiffness). In this regard, Figure 11 reports the simulated sensor response for six representative stiffness values (i.e., 0, 10, 40, 500, 1000 and 6000 MPa). The bone callus stiffness values of 0, 10 and 1000 MPa correspond to the beginning of the three main phases of healing, as indicated in Table 1. These results suggest that our capacitive sensor technology can detect pins displacements due to external load associated with different healing phases with a resolution comparable to that of the optical tracking system (0.25 mm). Moreover, the capacitive sensing module can be easily integrated with an external fixator, being, in the future, portable for longitudinal monitoring, thanks to the small dimensions of the single unit (L = 2.9 mm and W = 13.4 mm) and its signal conditioning electronics.

Comparison with other works can only be relevant if the frames are constructed with identical geometry and identical material properties of the components. Indeed, different researchers used different loading conditions, experimental setup and measurement targets. Although Burny et al. [13] declared the importance of considering the bone callus, since it can cause important variations in the load transmission, most of the previous studies did not consider the development of the bone callus in their analyses of the mechanical behaviour of the external frame [13]. In previous studies, the authors developed sensorized external fixators aiming to detect the deflection or the bending of the fixator body [8]. Moreover, these studies were mainly interested in evaluating the mechanical properties of different configurations of commercial external fixators, by using load cells [15,16], dial gauges [14,17] or strain gauges [13,38], rather than establishing a measurement of bone callus stiffness. Very few researchers worked on the sensorization of an external fixator to detect its displacement in relation to the healing time, such as Seide et al. [18] and Grasa et al. [19]; however, no advancements or applications of such technology were found in the recent literature. In conclusion, to the best of our knowledge, recent research in the context of bone fractures is still limited, and all of the previous works in this field employed measuring technologies, such as load cells, dial gauges and strain gauges. These measuring technologies present some disadvantage in view of future in-vivo applications: strain gauges are expensive, and more than one sensor is necessary to detect movements on different axes; load cells are cumbersome and not easily adaptable to any surface; dial gauges are also poor with regard to flexibility, and they can only provide linear measurements (not angular displacements). Below, a comparison between the current state-of-the-art technologies and the developed one is reported in Table 2.

Table 2 evaluates different KPIs, such as sensitivity, resolution, accuracy, time of response and costs. Sensor performances were tested in a non-structured environment without showing any significant drift. Parsing the achieved performances reported in Table 2, we can assert that the main advantages of the developed technology are its ability to be customizable according to the surface to be sensorized and to the demanded sensitivity range and resolution. Moreover, even if not strictly demanded by the application, the time of response of the capacitive sensor is below 10 ms, which allows researchers to use this technology for real-time applications. In summary, although the achieved accuracy is not comparable with that obtained with dials/strain gauges, the proposed capacitive-based measurement technology is suitable for our application at a reduced cost and with a higher flexibility and modularity with respect to the state-of-the-art.

We chose to implement capacitive technology, as it can overcome many of the mentioned issues, i.e., (1) it is cheap, (2) easily implemented, (3) adaptable to any non-regular surface, and furthermore (4) it has been proven to provide both proximity and pre-contact information [33]. Since we were able to work at pre-contact distances (i.e., 2 mm) in our measurement device, we exploited the exponential curve of response of the capacitive sensors to increase the resolution and the accuracy of the sensing technology. Moreover, the performance of a capacitive technology in detecting deformations and loads was already proved [43], paving the way for future orthopaedic applications. 

Further developments of the measurement device will be focused on the electronic circuit used for the system characterization to make it compatible, in the future, with portable configurations. In the current version, the stimulus generated by the external signal generator has a frequency of 30 kHz, with an amplitude of 1.3 *Vpp* and it can be easily substituted by a passive oscillator, which will generate a sinusoidal signal starting from a digital voltage generated by a commercial coin battery (3 V). Moreover, the power supply for the C/V converter (ADA4610-1, Analog Devices Inc., Norwood, MA, USA) will be generated by a dual boost DC-DC, which will act as a step-up voltage starting from the 3 V generated by the coin battery. Finally, a compact microcontroller embedding ADC channels and a Bluetooth module will be embedded into the measurement device with the purpose of converting and collecting the raw data coming from the sensors. All these electronic modifications will lead to the portable configuration of our measurement device. 

It is worth mentioning that the in-vitro models are intrinsically limited, due to their inability to reproduce the natural complex environment. In this study, we considered a standard tibia fracture with a well-defined gap of 20 mm, but many other challenges can occur in the real setting, such as a complex facture site, the presence of soft tissue, blood vessels and swelling (especially in the first phase of healing). Such elements are strictly dependent on the patient, the type and the site of the fracture, playing an additional and unknown role in the load distribution. So, authors are aware that the in-vivo translation of such a technology implies some complications that have to be faced in future in-vivo pilot experiments to assess the suitability of the proposed approach in the clinical scenario; however, the study represents a fundamental and propaedeutic technological step forward for the development of future smart medical measurement devices in the orthopaedic field. The measured parameters could be interpreted as an objective percentage of healing with respect to the specific patient and implant. Such an objective metric may support both surgeons and physiotherapists to assess the endpoint of healing and to set patient-specific rehabilitation protocols. The proposed methodology may overlap with the current approach and may support the diagnosis with a longitudinal, low-cost, non-invasive, and potentially even remote measurement of bone healing, allowing both a reduction in X-ray exposition and the integration of the current qualitative information.

## 5. Conclusions

In this study, we implemented a novel sensor technology, based on capacitive principles, which is able to provide: (a) a measurement of implant stability, and (b) a measurement of bone callus stiffness by recording the relative movements of the external fixator pins. Indeed, the monitoring of the positioning of the pins in the 3D space is fundamental in order to be certain that unwanted movements, not connected with the healing of the bone, do not occur before the measurement of callus stiffness. In this regard, a FEM model of the bone–external fixator system in the standard configuration, usually used to treat tibia fractures, was developed and verified. This model was used to derive the estimated pins displacements due to external compressive loads and the increasing stiffness of the bone callus, thus simulating the whole bone healing process. These specifications were used to develop an integrated capacitive sensor module, rigidly attached to the external pins to detect their relative 3D displacements. A five-sensor configuration was implemented to precisely monitor the positions of the pins in 3D space, whereas one sensor unit was employed to detect pins linear displacements under compressive loads for different degrees of bone callus stiffness. Our results demonstrate that the proposed configuration was able to: (a) detect changes in the position of the sensor supports in the 3D space, with a resolution of 0.5 mm and 0.5°, and (b) precisely monitor the pins displacements due to external loads, thus enabling an indirect measurement of bone stiffness.

The proposed sensor technology may have a potential impact in the context of bone fracture, offering a quantitative and objective tool to determine the progress and the endpoint of healing and to establish patient-specific rehabilitation protocols in order to positively influence and reduce the healing timeline.

## Figures and Tables

**Table 1:** Clinical indications for weight bearing during the bone healing time.

Healing Phases	Time Post Fracture	Type of Tissue	Callus Stiffness [Range, MPa]	External Load (Percentage of the Body Weight, %)
Inflammation/soft callus	Up to 3 weeks	Granulation tissue/cartilage	[0–10]	~30%
Soft callus/hard callus	From 3 to 6 weeks	Cartilage/woven bone	[10–1000]	~70%
Remodelling	From months to years	Woven bone/mature bone	[1000–6000]	100% (full body weight)

**Table 2:** Performance comparison of the proposed capacitive technology (last row) with respect to the current state-of-the-art. “N/A” is reported when the information is either too variable or not explicitly provided.

Tech.s	Resolution	Time of Response	Accuracy	Sensitivity	References
Dial Gauge	0.25 mm	N/A	10 µm	N/A	[39,40]
Strain Gauge	0.06 mm	N/A	2.6 µm	N/A	[19,20,21,22,23,24,25,26,27,28,29,30,31,32,33,34,35,36,37,38,39,40,41]
X-ray	0.2 mm	N/A	2.47 mm	N/A	[42]
**Capacitive**	**0.5 mm** **0.5°**	**5–10 ms**	**0.15 mm **	**[0–3.30 mm]**	**[33]**

## Data Availability

The datasets generated and/or analysed during the current study are available from the corresponding authors (A.S. and M.C.) on specific requests.

## Supplementary Materials

The following are available online at https://www.mdpi.com/article/10.3390/s21196694/s1, Figure S1—Comparison between the pins displacements calculated considering three axes and the pins displacement calculated considering only the longitudinal axis; Figure S2—Response of one single capacitive module with respect to movements in the 3D space.
