# Supplementary material for: A Novel Capacitive Measurement Device for Longitudinal Monitoring of Bone Fracture Healing
Source: Sensors (Basel). 2021 Oct 8;21(19):6694. doi: 10.3390/s21196694 (PMC8512850; doi:10.3390/s21196694)
Supplement: Supplementary file 1 [file sensors-21-06694-s001.zip › sensors-1377724-supplementary.pdf]

SUPPLEMENTARY MATERIALS

A Novel Capacitive Measurement Device for Longitudinal Monitoring of Bone Fracture Healing

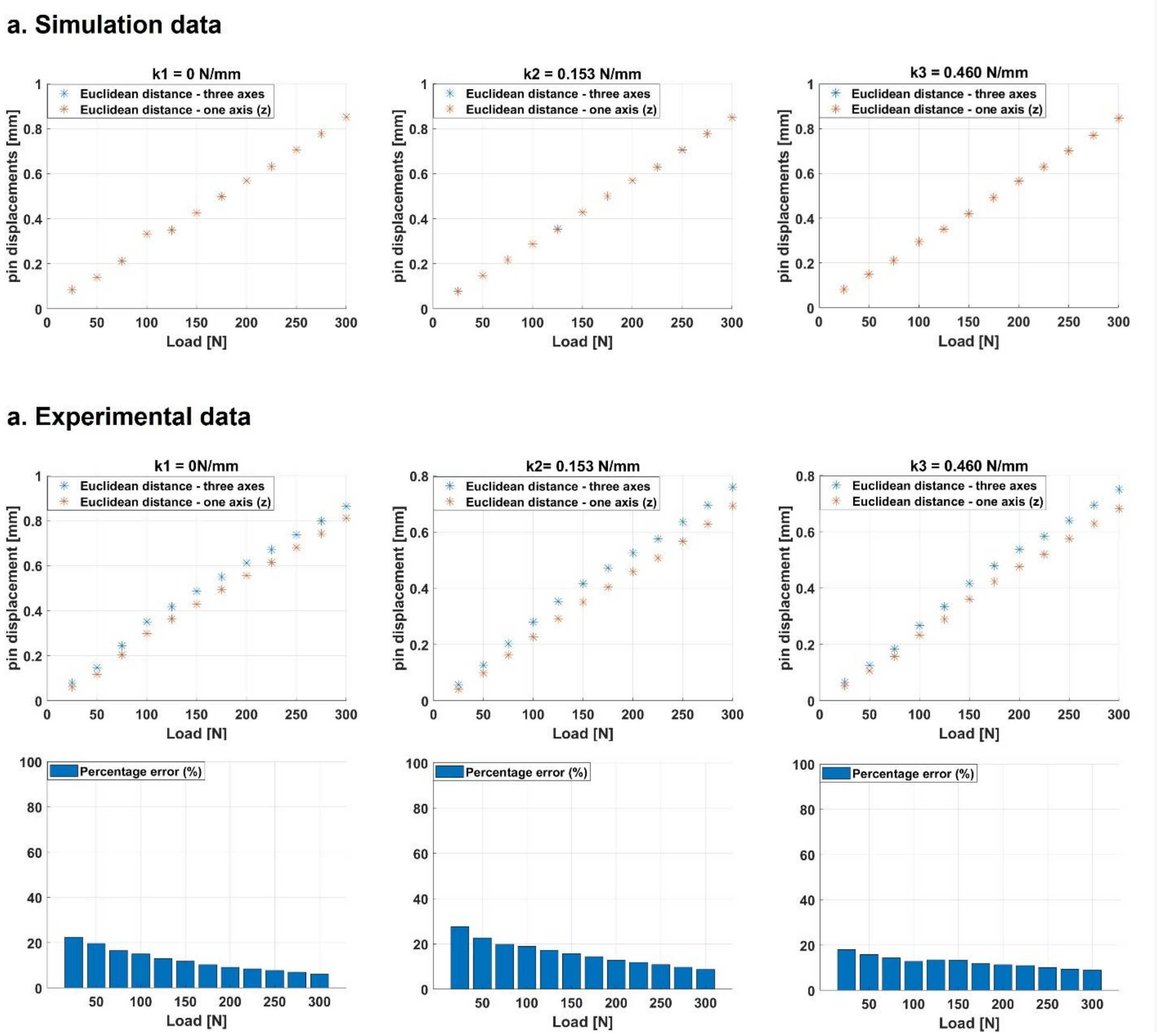

Figure S1 – Comparison between the pins displacements calculated considering three axes and the pins displacements calculated considering only the longitudinal axis. In this figure, the pins displacements calculated using the Euclidean distance on three axes and the Euclidean distance on the longitudinal axis were compared using the data from the simulation (a) and the data from the experiments (b) performed as described in Section 2.1 "Design and validation of a bone-external fixator model". No variations between the two distances were encountered when the simulation data were processed, while small errors between the two types of distances were found in case of experimental data, probably due to their high variability. However, the percentage errors between the Euclidean distance on the longitudinal axis and the Euclidean distance on three axes were modest (about 20%) for all the tested stiffness conditions (k_1, k_2 and k_3). Hence, the displacements of the pins in the transversal plane can be neglected in case of compressive loads and the movement can be considered completely axial with good approximation.

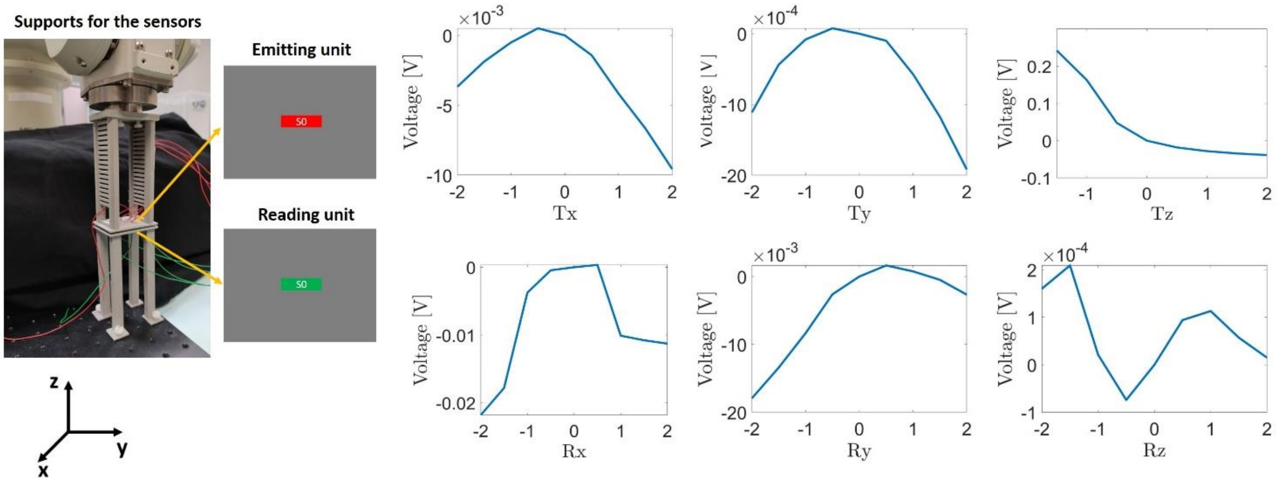

Figure S2 – Response of one single capacitive module with respect to movements in the 3D space. The support for the sensor was fixed to the end-effector of a robotic arm to provide all the possible movements in the 3D space (T_x, T_y, T_z, R_x, R_y and R_z). All the details about the experimental setup and the protocol are described in Section 3.3 "Design of a capacitive sensor technology to discriminate pins displacements" of the main manuscript. The results prove that one single module is able to detect the displacements along the longitudinal axis (T_z) taking into account also the direction of the movement (the voltage increases for negative movements, while decreases for positive movements). The displacements in the transversal plane (T_x, T_y, R_x and R_y) are reasonably detected by the single capacitive sensor unit; in particular, a decrease in sensor voltage is caused by both T_x and T_y and furthermore, the direction of the movement cannot be identified. On the other hand, the response of the single module with respect to the rotation around the longitudinal axis (R_z) is quite ambiguous and difficult to be interpreted. Hence, these results suggested that one single sensor is not enough to discriminate movements in the 3D space, and hence a combination of five sensors was exploited and tested for this purpose (for details, see result in figure 10 of the main manuscript).
